# Improved Minimum Squared Error Algorithm with Applications to Face Recognition

**DOI:** 10.1371/journal.pone.0070370

**Published:** 2013-08-06

**Authors:** Qi Zhu, Zhengming Li, Jinxing Liu, Zizhu Fan, Lei Yu, Yan Chen

**Affiliations:** 1 Bio-Computing Center, Harbin Institute of Technology Shenzhen Graduate School, Shenzhen, China; 2 School of Optical-Electrical and Computer Engineering, University of Shanghai for Science and Technology, Shanghai, China; 3 Key Laboratory of Network Oriented Intelligent Computation, Shenzhen, China; 4 Guangdong Industrial Training Center, Guangdong Polytechnic Normal University, Guangzhou, China; 5 College of Information and Communication Technology, Qufu Normal University, Rizhao, China; 6 School of Basic Science, East China Jiaotong University, Nanchang, China; 7 School of Urban Planning and Management, Harbin Institute of Technology Shenzhen Graduate School, Shenzhen, China; 8 Shenzhen Sunwin Intelligent Co., Ltd., Shenzhen, China; UC Davis School of Medicine, United States of America

## Abstract

Minimum squared error based classification (MSEC) method establishes a unique classification model for all the test samples. However, this classification model may be not optimal for each test sample. This paper proposes an improved MSEC (IMSEC) method, which is tailored for each test sample. The proposed method first roughly identifies the possible classes of the test sample, and then establishes a minimum squared error (MSE) model based on the training samples from these possible classes of the test sample. We apply our method to face recognition. The experimental results on several datasets show that IMSEC outperforms MSEC and the other state-of-the-art methods in terms of accuracy.

## Introduction

The minimum squared error based classification (MSEC) is sound in theory and is able to achieve a high accuracy [1], [2]. It has been proven that for two-class classification MSEC is identical to linear discriminant analysis (LDA) under the condition that the number of training samples approximates the infinity [1], [2]. In addition, MSEC can be applied to multi-class classification by using a special class label matrix [3]. Various improvements to MSEC such as orthogonal MSEC [4] and kernel MSEC [5]–[8] have been proposed. The MSEC has been applied to a number of problems such as imbalanced classification [7], palm-print verification [9], low-rank representation [10], [11], super-resolution learning [12], image restoration [13], and manifold learning [14].

In recent years, representation based classification (RC) method [15]–[18] has attracted increasing attention in pattern recognition. The main difference between RC and MSEC is that RC tries to use the weighted sum of all the training samples to represent the test sample, whereas MSEC aims to map the training samples to their class labels. RC can be categorized into two types. The first type is the so-called sparse representation method (SRM) such as the methods proposed in [19], [20]. The goal of SRM is to simultaneously minimize the 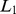 norm of the weight vector and the representation error that is the deviation between constructed sample and test sample. The second type is the so-called non-sparse representation method such as the methods proposed in [21]–[26]. The goal of the non-sparse representation method is to simultaneously minimize the 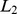 norm of the weight vector and the representation error. The non-sparse representation method has a closed-solution and is usually more computationally efficient than SRM [21].

In this paper, we focus on multi-class classification problem and propose an improved minimum squared error based classification (IMSEC) method. The basic idea of IMSEC is to select a subset of training samples that are similar to the test sample and then build the MSE model based on them. The advantage of the IMSEC is that it seeks the optimal classifier for each test sample. However, MSEC categorizes all the test samples based on a unique classifier. Therefore IMSEC has better performance than MSEC.

### The minimum Squared Error Based Classification for Multi-class Problems

Suppose that there are 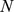 training samples from 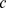 classes. Let the 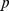-dimensional row vector 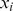 denote the 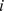-th training sample, where 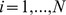. We use a 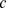-dimensional row vector 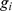 to represent the class label of the 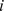-th training sample. If this sample is from class 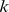, the 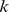 **-**th entry in 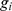 is one and the other entries are all zeroes.

If a mapping 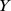 can approximately transform each training sample into its class label, we have
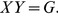
(1)where 
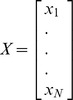
, 
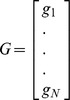
. Clearly, 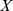 is an 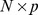 matrix, 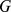 is an 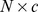 matrix, and 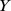 is a 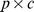 matrix that is to be solved. As Eq. (1) cannot be directly solved, we convert it into the following equation:
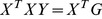
(2)


If 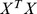
is non-singular, 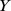 can be solved by
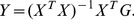
(3)


In general, we use 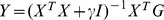 to obtain a stable numerical solution, where 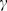 and 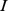 denote a small positive constant and the identity matrix, respectively.

Finally, we classify a test sample 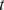 as follows: the class label of 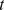 is predicted using 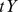, and then the Euclidean distance between 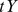 and the class label of each class is calculated, respectively. The class label of the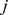-th class is a row vector whose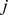-th element is one and whose other elements are all zeros (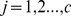). Among the 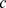 classes, if 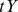 is closest to the 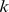-th class, then 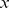 is classified into the 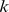-th class.

### The Algorithm of Improved Minimum Squared Error Based Classification

Suppose the 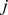-th class has 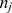 training samples. Let 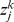 be the 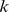-th training sample of the 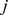-th class, where 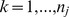,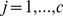. The algorithm of IMSEC has the following three steps.

#### Step 1

Determine 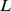 possible classes of the test sample, where 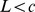. First, the test sample 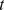 is represented as a weighted sum of the training samples of each class, respectively. For the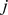-th class, it is assumed that 
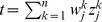
 is approximately satisfied. 
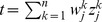
 can be rewritten as 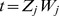, where 
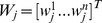
 and 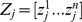. Then we have 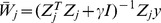. 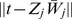 is the representation error between the training samples of 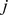-th class and the test sample. The 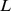 classes that have the smallest 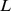 representation errors are determined, and they are referred to as base classes.

#### Step 2

Use the base classes to establish the following MSE model
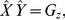
(4)where 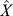 is composed of all the training samples of the base classes, and 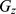 is composed of the class labels of these training samples. 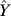 is computed using 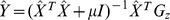. 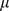 and 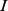 are a small positive constant and identity matrix, respectively.

#### Step 3

Exploit 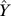 and 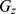 to classify the test sample 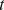. The class label of this test sample can be predicted by using 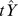. Calculate the Euclidean distance between 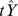 and the class label of each base class, respectively. Let 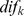 denote the distance of the between 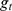 and the class label of the 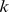-th class. If 
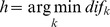
, then test sample 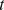 is assigned into the 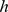
- th class.

### Analysis of the Proposed Method

The proposed method and the MSEC have the following differences. MSEC attempts to obtain a unique model for all the test samples, whereas the proposed method constructs a special MSE for each test sample. MSEC tries to minimize the mean square error between the predicted class labels and the true class labels of the training samples. That means MSEC is capable of mapping the training samples to the correct class labels. However, this does not imply that the model of MSEC can map the test sample to the correct class label accurately. Since the test sample and the training samples that are “close” to the test sample have the similar MSE models, it can be expected that IMSEC performs better in mapping the test sample to the correct class label than CMSE.

The proposed method works in the way of coarse-to-fine classification. In detail, step 1 of the proposed method indeed roughly identifies the possible classes of the test sample. Step 2 of the proposed method assigns the test sample into one of the possible classes. For the complicated classification problem, the way of coarse-to-fine classification is usually more effective than the way in one step [27]–[29].

It is worth pointing out that the proposed method is different from CRC [21] and linear regression based classification (LRC) [30]. The proposed method tries to establish a model to map the training samples to their true class labels, whereas CRC uses the weighted combination of all the training samples to represent the test sample, and LRC uses the class-specific training samples to represent the test sample. Moreover, when classifying a test sample, the proposed method and LRC need to solve one and 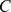 MSE models, respectively, where 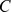 is the number of the classes. As a result, the proposed method is more efficient than LRC.

## Experiments

### A. Ethics Statement

Some face datasets were used in this paper to verify the performance of our method. These face datasets are publicly available for face recognition research, and the consent was not needed. The face images and the experimental results are reported in this paper without any commercial purpose.

### B. Experimental Results

Face recognition has become a popular pattern classification task. We perform the experiments on ORL, FERET and AR face databases. Our method, CMSE, CRC, SRC, Eigenface [31], Fisherface [32], Nearest Neighbor Classifier (1-NN), 2DPCA [33], Alternative-2DPCA [34], 2DLDA [35], Alternative-2DLDA [36] and 2DPCA+2DLDA [37] were tested in the experiments. Before implementing each method, we converted every face image into a unit vector with the norm of 1. When CRC was implemented, the regular parameter was set to 0.001. In Eigenface method, we used the first 50, 100…, 400 Eigenfaces for feature extraction, respectively, and reported the lowest error rate. In the 2D based subspace methods, including 2DPCA, Alternative-2DPCA, 2DLDA, Alternative-2DLDA and 2DPCA+2DLDA, the number of the projection axes was set to 1,2,…,5, and the lowest error rate was reported.

**Figure 1:**
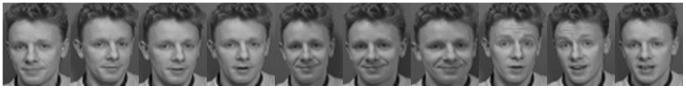
The face images of one subject in the ORL database.

In the ORL database, there are 40 subjects and each subject has 10 different images. For some subjects, the images were taken at different times, varying the lighting, facial expressions (open/closed eyes, smiling/not smiling) and facial details (glasses/no glasses). All the images were taken against a dark homogeneous background with the subjects in an upright, frontal position (with tolerance for some side movement). Each face image contains 92
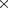
112 pixels, with 256 grey levels per pixel [38]. We resized each face image into a 46 by 56 matrix. Figure 1 shows the face images of one subject in the ORL database. We took the first three, four, five and six face images of each subject as training images and treated the others as test images, respectively. In our method, 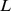 was set to 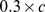.

**Figure 2:**
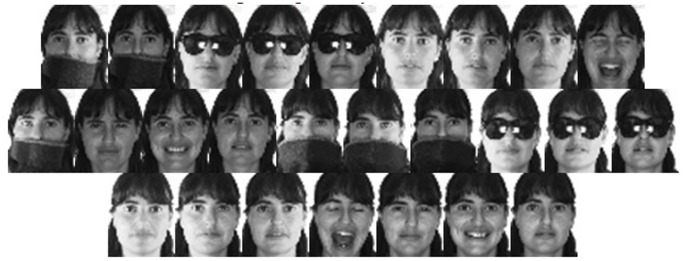
The face images of one subject in the AR database.

For AR face database, we used 3120 gray face images from 120 subjects, each providing 26 images [39]. These images were taken in two sessions and show faces with different facial expressions, in varying lighting conditions and occluded in several ways. Figure 2 shows the 26 face images of one subject in the AR database. We took the first four, five, six, seven and eight face images of each subject as training images and treated the others as test images, respectively. In our method, 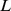 was set to 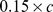.

**Figure 3:**
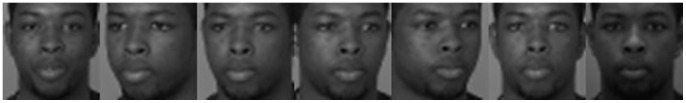
Some face images from the FERET database.

A subset of the FERET face database is used to test our method. This subset includes 200 subjects, and each subject has 7 images. It is composed of the images whose names are marked with two-character strings: ‘ba’, ‘bj’, ‘bk’, ‘be’, ‘bf’, ‘bd’, and ‘bg’. This subset involves variations in facial expression, illumination, and pose [40]. The facial portion of each original image was cropped to form a 40
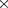
40 image. Figure 3 shows some face images from the FERET database. We took the first five and six face images of each subject as training images and treated the others as test images, respectively. In our method, 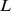 was set to 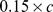.

Tables 1, 2 and 3 show the classification error rates of the methods on the ORL, AR and FERET databases, respectively. We can observe that our method always obtains the lowest classification error rate. In other words, our method can achieve the desirable classification result.

**Table 1:** Rates of classification errors of the methods on the ORL database (%).

Number of the originaltraining samplesper class	3	4	5	6
The proposed method	11.07	5.83	4.50	1.87
CMSE	13.93	7.92	7.50	3.75
CRC	15.36	9.17	8.00	5.63
SRC	19.29	15.00	14.50	11.87
Eigenface	26.07	20.00	14.00	10.00
Fisherface	23.01	22.64	23.29	9.08
1-NN	20.36	15.00	14.00	8.75
2DPCA	14.29	11.25	9.50	3.75
Alternative-2DPCA	13.93	10.42	8.50	3.75
2DLDA	11.79	7.92	9.50	4.37
Alternative-2DLDA	17.50	13.75	13.50	4.37
2DPCA+2DLDA	16.07	12.50	10.00	4.37

**Table 2:** Rates of classification errors of the methods on the AR database (%).

Number of the original training samples per class	4	5	6	7
The proposed method	25.27	23.29	22.96	20.92
CMSE	27.92	24.88	25.87	25.48
CRC	29.89	28.02	29.71	28.90
SRC	41.97	43.41	34.04	29.78
Eigenface	41.78	47.66	24.79	26.05
Fisherface	44.17	40.71	25.13	23.89
1-NN	37.69	39.40	25.87	25.04
2DPCA	40.38	41.87	30.83	31.36
Alternative-2DPCA	40.23	40.87	30.63	31.54
2DLDA	50.68	52.22	35.33	33.25
Alternative-2DLDA	54.09	55.83	41.96	36.40
2DPCA+2DLDA	35.53	37.90	26.42	28.03

**Table 3:** Rates of classification errors of the methods on the FERET database (%).

Number of the originaltraining samplesper class	5	6
The proposed method	19.00	3.50
CMSE	29.25	7.50
CRC	38.75	29.50
Eigenface	37.00	36.00
Fisherface	47.50	61.00
SRC	59.25	52.50
1-NN	28.50	27.00
2DPCA	35.25	49.50
Alternative-2DPCA	36.00	50.00
2DLDA	29.25	25.25
Alternative-2DLDA	29.25	31.50
2DPCA+2DLDA	30.50	35.00

### Conclusions

The proposed method, i.e. IMSEC, establishes a special MSE model for each test sample. When building the classification model, IMSEC uses only the training samples that are close to the test sample. Theoretical analyses were presented to explore the properties of IMSEC. Compared with MSEC that classifies all the test samples based on a unique model, IMSEC can perform better in classifying the test samples. We tested the proposed method on three face datasets. The experimental results clearly demonstrated that IMSEC outperforms MSEC and the other state-of-the-art methods.
